# 299 Colonizer or Culprit? Significance of Stenotrophomonas maltophilia in Urine Cultures

**DOI:** 10.1017/ash.2026.10655

**Published:** 2026-06-23

**Authors:** Devin Ray, Grace St. Cyr, Logan Grimes

**Affiliations:** 1 The Children’s Hospital of Philadelphia; 2 Children’s Hospital of Philadelphia

## Abstract

**Background:** Due to concern for ongoing colonization and substantial risks posed by horizontal transmission of RGN in acute care settings, it is common practice in many institutions to employ indefinite CP once a patient tests positive for resistant bacteria. However, it is important to recognize the adverse environmental and patient care costs of CP which has led institutions to consider structured CP discontinuation policies. Emerging evidence supports implementation of practices to safely discontinue CP in select patients following a defined period without subsequent positive cultures. Despite this, standardized methods to identify eligible patients remain unexplored. Objective: As a part of a larger study to evaluate our institution’s adopted policy of contact precaution (CP) discontinuation for resistant gram negative organisms (RGNs) after one year of no subsequent positive cultures, we aimed to assess the utility of a retrospective audit of medical records as a strategy to identify patients eligible for CP discontinuation. **Methods:** A retrospective chart review of patients with identified RGNs in the Children’s Hospital of Philadelphia (CHOP) electronic medical record (EMR) system from May 2021 to November. In concordance with the internal policy established November 202 recommending removal of contact precautions after one year without subsequent RGN culture positivity, each chart was assessed for the date and organism of the initial infection as well as last positive culture to determine eligibility for CP removal. Patients with carbapenem resistant organisms or those with cystic fibrosis were excluded from the review. **Results:** Of the/of the identified patients were determined to be eligible for de-labeling. The time required to determine eligibility status in the EMR was approximately 1 minute per patient totaling around 2.5 hours of review. **Conclusion:** This review identified a significant proportion of RGN-positive patients for whom standing CP were not removed in real time, allowing for an efficient update of CP status to align with hospital policy. Removal of CP not only alleviates a significant environmental and financial burden but also could contribute to a more positive patient/family experience during the hospital stay. The results of this study suggest that employing prospective plans for chart audits as CP discontinuation policies are modified would improve resource utilization.

